# Sprouted Innervation into Uterine Transplants Contributes to the Development of Hyperalgesia in a Rat Model of Endometriosis

**DOI:** 10.1371/journal.pone.0031758

**Published:** 2012-02-21

**Authors:** Stacy L. McAllister, Natalia Dmitrieva, Karen J. Berkley

**Affiliations:** Program in Neuroscience, Florida State University, Tallahassee, Florida, United States of America; University of Jaén, Spain

## Abstract

Endometriosis is an enigmatic painful disorder whose pain symptoms remain difficult to alleviate in large part because the disorder is defined by extrauteral endometrial growths whose contribution to pain is poorly understood. A rat model (ENDO) involves autotransplanting on abdominal arteries uterine segments that grow into vascularized cysts that become innervated with sensory and sympathetic fibers. ENDO rats exhibit vaginal hyperalgesia. We used behavioral, physiological, and immunohistochemical methods to test the hypothesis that cyst innervation contributes to the development of this hyperalgesia after transplant. Rudimentary sensory and sympathetic innervation appeared in the cysts at two weeks, sprouted further and more densely into the cyst wall by four weeks, and matured by six weeks post-transplant. Sensory fibers became abnormally functionally active between two and three weeks post-transplant, remaining active thereafter. Vaginal hyperalgesia became significant between four and five weeks post-transplant, and stabilized after six to eight weeks. Removing cysts before they acquired functional innervation prevented vaginal hyperalgesia from developing, whereas sham cyst removal did not. Thus, abnormally-active innervation of ectopic growths occurs before hyperalgesia develops, supporting the hypothesis. These findings suggest that painful endometriosis can be classified as a mixed inflammatory/neuropathic pain condition, which opens new avenues for pain relief. The findings also have implications beyond endometriosis by suggesting that functionality of any transplanted tissue can be influenced by the innervation it acquires.

## Introduction

Endometriosis is a painful disorder defined by extrauteral endometrial growths [1]–[3]. Pains include severe dysmenorrhea, dyspareunia (vaginal hyperalgesia), and chronic pelvic pain. Endometriosis often co-occurs with other painful disorders such as interstitial cystitis/painful bladder syndrome, irritable bowel syndrome, migraine and other headaches, and more. Most gynecologists characterize endometriosis as an “enigma,” mainly because pain symptoms are unrelated to the amount of ectopic growth, and the pain is difficult to treat. New approaches are badly needed. Although about ten years ago it had been noted that pain can be more severe in patients whose ectopic growths are located near or in richly innervated anatomical sites (rectovaginal septum, uterosacral ligaments) than in patients whose growths invaded less densely innervated tissue (peritoneum and/or ovaries) [4], [5], scant attention was paid to this finding regarding nerves; research and drug development remained focused on the ectopic growths and their immediate external environment (e.g., peritoneal fluid and local inflammation) as the source of the pain.

A rat model of endometriosis is created by autotransplanting on abdominal arteries small pieces of uterus (ENDO). These transplants become vascularized and form cysts [6]. As a control, small pieces of fat are similarly autotransplanted (shamENDO), but no cysts form. As in women with endometriosis, rats with established ENDO (but not shamENDO) exhibit vaginal hyperalgesia [7], and the amount of cystic growth does not correlate with hyperalgesic severity [8], [9].

Our group discovered that ectopic growths harvested from ENDO rats and women with established endometriosis develop their own C-fiber (sensory afferent) and sympathetic (autonomic efferent) nerve supply. The supply is derived from nerve fibers innervating nearby territories that sprout branches into the growths [10], [11]. This discovery suggests that, rather than the growths alone, it is the ectopic growth's own innervation that is a major contributor to the maintenance and modulation of pain in established endometriosis [12]. It remains unknown, however, whether this sprouted and thereby abnormal innervation contributes to the initial development of endometriosis-associated pain. If so, painful endometriosis, now recognized as an inflammatory condition [3], could also be considered within the realm of neural dysfunction, i.e., a mixed inflammatory and neurogenic pain, which would alter clinical approaches to the pain.

Here, using the rat model, we tested the hypothesis that nerve fibers sprouting into the transplants after surgery underlie the development of vaginal hyperalgesia. If so, the time course of development of abnormally functioning innervation should precede and then parallel development of the hyperalgesia. Furthermore, removal of the cysts before significant hyperalgesia and stable innervation develops should prevent the hyperalgesia from occurring. These predictions were tested in four studies. Behavioral and immunohistochemical methods were used to assess, respectively, the time course of development of vaginal hyperalgesia and the cysts' sensory and sympathetic innervation after transplant surgery. To determine when the nerve fibers become abnormally functionally active after they appear in the cysts, the extravasation of Evans Blue dye (EB) was assessed in nerve-intact compared with denervated cysts at different times after transplant. This method relies on the fact that activity of C-fibers produces the neurogenic contribution to EB extravasation [13] and that activity in sympathetic fibers can also contribute [14]. Therefore, abnormal nerve fiber activity in the cysts is indicated when denervation reduces EB extravasation. Finally, behavioral methods were used to assess the effects of removing the cysts before they acquired functional innervation on transplant-induced vaginal hyperalgesia. Our findings strongly support the hypothesis that the invasion of the cysts by sensory and sympathetic fibers is a major contributor to the development of endometriosis-associated pain, which suggests an aggressive clinical strategy of prevention.

## Results

### Vaginal hyperalgesia develops four weeks post-transplant and stabilizes six to eight weeks post-transplant

**Figure 1:**
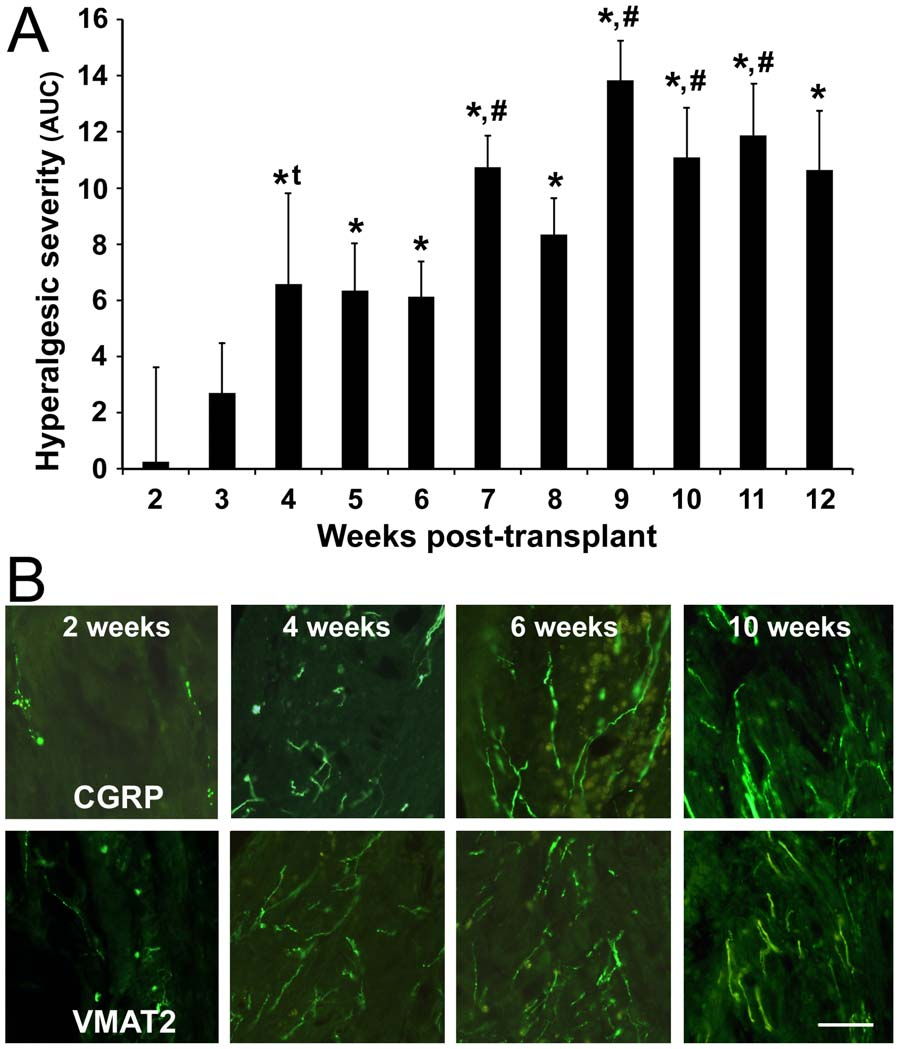
A behavioral study of the development of vaginal hyperalgesia and an immunohistochemical study of the development of cyst innervation after transplant surgery. Note that sensory and sympathetic fibers appear within the cysts two weeks before significant hyperalgesia develops. *A*. Hyperalgesic severity (post-transplant AUC minus baseline AUC) at different times after transplant surgery in the same rats. Error bars are ± SEM (*n* = 18). *, differs from two weeks; #, differs from six weeks, *p*<0.05. For the 4 week time point, *t = 0.058. *B*. Photomicrographs of sensory (CGRP-positive and sympathetic (VMAT2-positive) fibers in the cysts labeled at two, four, six, and ten weeks after transplant surgery (n = 3–5 rats/survival time). Calibration bar is 50 µm for all images.

From one to three weeks after transplant surgery, the probability of escape responses to vaginal distention did not differ significantly from baseline responses; i.e., no significant changes from baseline nociception had occurred (Fig. 1A). By four weeks after transplant surgery, however, responses tended to differ from baseline, becoming significantly different by five weeks. The hyperalgesia then increased to stabilize after 6 weeks.

### Sensory and sympathetic innervation of the cysts appears two weeks post-transplant and attains mature appearance by six weeks post-transplant (Figure 1B)

Immunofluorescence staining with antibodies to CGRP and VMAT2 revealed that both sensory and sympathetic neurites first appeared inside cysts two weeks post-transplant surgery; i.e., none were observed inside cysts harvested one week after transplant. The neurites at two weeks post-transplant were observed around blood vessels entering the hilus at the outer wall of the cysts, and had a rudimentary, mostly punctate appearance. By four weeks post-transplant, the characteristics of both sensory and sympathetic neurites changed their appearance so that they were no longer punctate, but instead branched and looked similar to fibers in established cysts. In addition, the fiber density increased dramatically and populated the entire cyst wall, including myometrial and endothelial layers. Both types of fibers were densest in the hilus area. By six weeks after transplant, the appearance of both types of fibers was similar to that at four weeks, with a slight increase in density and similar to that at ten weeks post-transplant. Overall, fiber characteristics at six weeks post-transplant were virtually identical to those previously observed in established cysts [10].

### Cyst innervation becomes functional approximately three weeks post-transplant (Figure 2)

**Figure 2:**
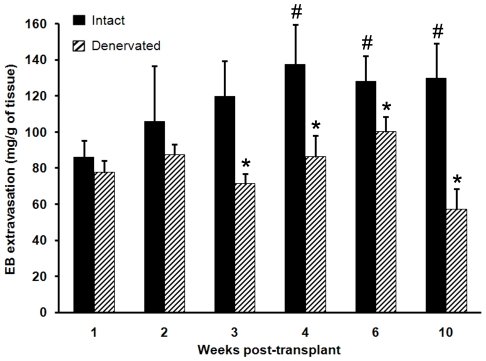
Evans Blue dye extravasation in intact and denervated cysts harvested at different times after transplant surgery. Note that the cysts' sensory innervation becomes functional (i.e., contributes to EB dye extravasation) three weeks after transplant surgery, which is one week before the hyperalgesia becomes significant (Fig. 1A). *, differs from intact at the same time period, *p*≤0.05. Within the Intact group: #, differs from the one-week time period, (*p*≤0.05). Error bars are ± SEM (*n* = 8–12/group).

EB dye extravasation into intact cysts increased progressively after transplant surgery (F_5,67_ = 2.90, *p* = 0.02) and became significantly different at four weeks post-transplant surgery compared with one week post-transplant (*p*<0.05). To estimate when neurogenic activity contributed to this extravasation, the extravasation in intact cysts was compared with the extravasation in cysts that had been denervated before the dye was injected. In other words, a neurogenic component would be revealed when denervation produced a reduction in extravasation. Of importance is that, whereas there was a gradual increase in dye extravasation in the intact cysts, there were no significant differences over time after transplant surgery in the denervated cysts (F_5,65_ = 0.77, *P* = 0.57). The differences between the intact and denervated cysts became significant three weeks after transplant surgery, and remained that way through ten weeks after transplant. In other words, sensory fibers became abnormally active (produced neurogenic activity) approximately three weeks after surgery.

### Removing cysts before cyst innervation becomes active, but not sham cyst removal, prevents subsequent hyperalgesia from developing

**Figure 3:**
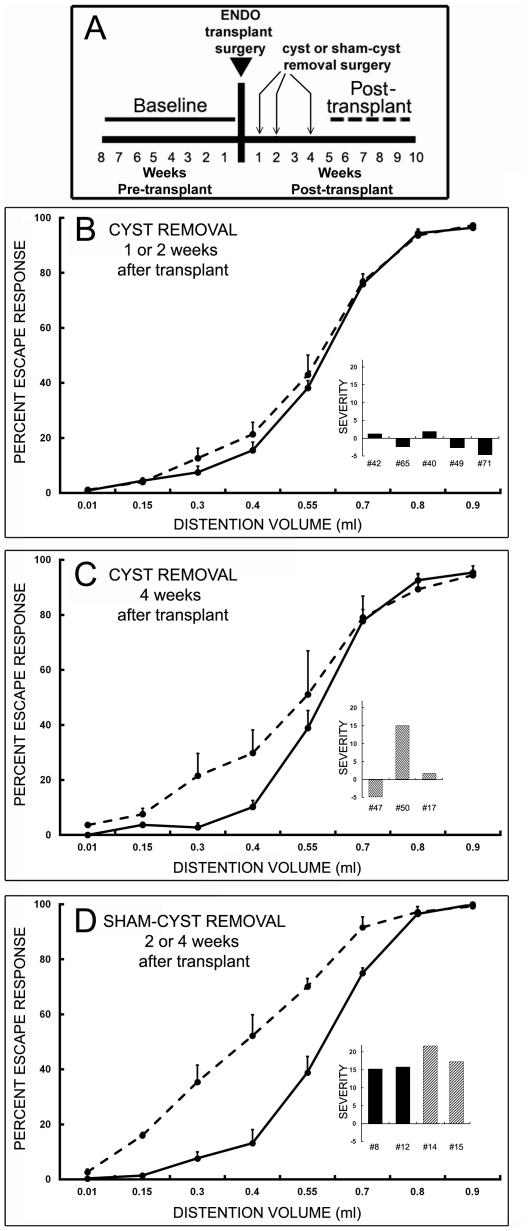
Effect of cyst removal or sham-cyst removal performed before the cysts acquire mature innervation on the development of vaginal hyperalgesia. ***A***. This diagram depicts the time-course of experimental testing (baseline, post-transplant) and surgical procedures (1, 2 or 4 weeks after transplant). **B**–**D.** The graphs show percent escape response as a function of vaginal distention volume before (baseline, solid line) and 5–10 weeks after transplant surgery (dashed line). Error bars are ± SEM. The inset bar graphs depict the severity of hyperalgesia individually for each rat in that group (as in Fig. 1). Solid bars indicate cyst removal or sham-cyst removal at 1 or 2 weeks post-transplant; the hatched bars indicate cyst removal or sham-cyst removal at 4 weeks post-transplant. **B.** Pre-innervation cyst removal (1 or 2 weeks post-transplant) prevents the development of hyperalgesia (n = 5). **C.** When cyst removal is performed just after innervation has become active, but before innervation is mature; i.e., 4 weeks post-transplant, the development of hyperalgesia is prevented in two of three rats. **D.** In contrast, after sham-cyst removal, regardless of when the surgery is performed relative to the innervation (either 2 or 4 weeks after transplant), hyperalgesia develops in all rats (n = 4).

Figure 3 shows the effects of cyst removal or sham cyst removal during the 4 week period after transplant (when the cysts are being invested with nerve fibers) on the subsequent development of hyperalgesia. Overall, when cysts were removed any time during this period (n = 8), significant vaginal hyperalgesia failed to develop (F_1,14_ = 0.68, *P* = 0.80). This overall failure occurred either when cysts were removed two weeks or less after transplant surgery (Fig. 3B, n = 3; [F_1,8_ = 0.28, *P* = 0.61]) or when cysts were removed later at 4 weeks after transplant surgery (Fig. 3C; [F_1,4_ = 0.43, *P* = 0.55]), although one of the 3 rats in this later group did exhibit hyperalgesia. In contrast, when a sham cyst-removal surgery was performed from 2–4 weeks post-transplant time in a different group of rats, significant vaginal hyperalgesia developed in all rats regardless of when the sham-cyst removal surgery had been done (Fig. 3D, n = 4; [F_1,6_ = 27.1, *P* = 0.002]).

## Discussion

Our studies show that a three-part sequence of events occurs after transplant surgery in the rat model. Initially, two weeks after transplant, the endometrial cysts acquire a rudimentary sensory and sympathetic innervation. A week later, three weeks post-transplant, this abnormal innervation becomes functional; i.e., it is capable of contributing to neurogenic inflammation. By four to five weeks, at which time the cysts are populated by sensory and sympathetic nerve fibers, the hyperalgesia becomes significant. Finally, removing the cysts before they acquire functional innervation prevents the hyperalgesia from developing, whereas a sham surgery does not. These results provide strong support for the hypothesis that the cysts' sensory and sympathetic innervation underlies the development of vaginal hyperalgesia after transplant surgery.

These findings relate to previous studies in the rat model that indirectly suggest that, after the cysts stabilize, their sensory and sympathetic innervation influences the severity of transplant-associated hyperalgesia. Thus, it was found that, after the cysts have stabilized (i.e., more than 8 weeks post-transplant), vaginal nociception varies with estrous stage, with hyperalgesia present in proestrus but absent the next day in estrus [7]. Later, it was found that, at that >8 week post- transplant time, there are parallel proestrous-to-estrous reductions in both the density of the cyst's sympathetic innervation and the cyst's content of growth factors that likely affect activity of sensory C-fibers [15]. Similarly, at that time, when a failure to remove all cysts during surgery produces an increase in hyperalgesia, that increase is accompanied by an increase in the density of sympathetic fibers in the cysts [9]. Together, these previous and our current results indicate that there is a continuous and, importantly, a changing role of sensory and sympathetic innervation of the cysts. Initially the innervation contributes to the development of transplant-induced vaginal hyperalgesia. Later, after the innervation is established, the innervation contributes to modulation of its severity. Although there are no clinical data yet available that address the former component (neural sprouting into ectopic growths during early stages of pain development), evidence is accumulating in women to support the latter component (neural modulation of pain once endometriosis is established) [12], [16].

Here we showed in the rat that sprouting of C-fibers and sympathetic fibers occurs in response to the initial pathology of the transplants. Such sprouting is known to be associated with an increase in the background and evoked activity of these fibers; that is, fibers that develop sprouts become sensitized [17], [18], which contributes to protein extravasation [13]. This increased nociceptive activity in turn produces changes in the excitability of spinal neurons, a phenomenon called central sensitization [19], [20], which can expand from spinal segments where the fibers enter to more remote regions [21]. In other words, the initial neural pathophysiology (sprouting and abnormal nerve activity) triggered by the ectopic growths can, over time, induce central sensitization, leading to chronic pain and co-morbidity [20]. Importantly, central sensitization can eventually become self-sustained and independent of peripheral pathology so that, in some women with endometriosis, surgical removal of the ectopic growths would fail to alleviate pain [3].

Insofar as data in rats can help us understand mechanisms that contribute to pain in women with endometriosis (see table IV in ref. 3), our findings here suggest that painful endometriosis in women, already considered an inflammatory condition [3], might benefit from being additionally classified as a neuropathic pain condition. Neuropathic pain has recently been formally defined by the International Association for the Study of Pain as “pain caused by a lesion or disease of the somatosensory nervous system” (http://www.iasp-pain.org/AM/Template.cfm?Section=Pain_Defi…isplay.cfm&ContentID = 1728#Neuropathicpain, accessed October 12, 2011). Although a neuropathic pain classification is usually applied to painful skin or musculoskeletal conditions that involve direct damage to peripheral or central neural structures, it has been argued that visceral C-fibers that are sensitized in association with an initial inflammation “may become a source of neuropathic pain” [22]. Thus, for example, a neuropathic classification has recently been effectively applied to pain associated with visceral disorders such as pancreatitis, in which abnormal nerve sprouting, like that associated with endometriosis, plays a substantial role in the development of pain [23].

Our findings here clearly put an emphasis on prevention. There is an astonishingly high prevalence of up to 90% of adolescent girls who suffer from dysmenorrhea, with 15–20% reporting it as severe [24]–[26]. A significant portion of this latter group is likely to have endometriosis [3]. Recent brain imaging studies have shown that moderate-to-severe dysmenorrhea is associated with significant brain dysfunction and abnormalities in the hypothalamic–pituitary–adrenal (HPA) axis (a reduction in cortisol levels) [3], [27]–[30]. Furthermore, and importantly, these same studies also show that the longer the history of the pain, the more severe the effects on the brain and the HPA axis. Thus, one immediate consequence of reframing endometriosis as a neuropathic/inflammatory pain condition would be to reinforce the urgency of developing strategies for earlier diagnosis of endometriosis [30], [31] as well as treatment of its associated pain (e.g., dysmenorrhea) before that pain can become chronic and other painful co-morbid conditions arise. Such classification would also bring under consideration how therapeutic strategies now in use or under development for neuropathic pain might be applied to painful or potentially painful endometriosis [32], [33].

Besides endometriosis, our results have relevance to another, seemingly unrelated clinical situation: tissue/organ transplantation. Due to their removal from the donor, all donor tissues and organs are denervated before transplantation. The question therefore arises as to the importance of re-innervation for the health and proper functioning of the transplanted organ, as well as the possibility, raised here, that such re-innervation could give rise to neuropathic-type pain. The topic has not been extensively studied. Some evidence exists for re-innervation of the auto-transplanted heart and syngeneically-transplanted kidney in experimental animals [34], [35]. Most studies in humans of re-innervation of transplanted organs (necessarily allogenic) generally indicate that there is limited sympathetic fiber re-innervation by a year or more after transplant [36], [37]. Debate about the importance of this re-innervation for healthy organ functioning continues, but is generally supportive [38]. Some findings also suggest limited sensory fiber re-innervation, as evidenced by the fact that pain can be provoked by cardiac ischemia in heart transplant patients [39] or lobeline injections in lung transplant patients [40]. The issue of whether either chronic pain or co-morbidity develops after transplant, is, however, complicated by the lifetime cocktail of neurotoxic immunosuppressant and other drugs that patients must take [41]. Nevertheless, the very limited evidence so far seems to suggest that re-innervation may be a more significant factor for uterine transplants or ectopic endometrial growths than it is for other organs. One contributing feature may be the hormonal susceptibility of the innervation of the uterine or ectopic endometrial growths. Thus, sympathetic innervation density of the ectopic uterine growths varies with estrous stage [15]. Furthermore, others have shown in both rodents and humans that innervation of the healthy uterus also undergoes anatomical changes during the ovarian cycle as well as significant changes during pregnancy [42]. Despite considerable research on hormonal influences on the physiological functioning of other organs, however, little is known about such anatomical changes in innervation of other tissues. Together, these considerations indicate the potential translational value of future studies aimed at comparing the re-innervation and consequences of that re-innervation in different transplanted organs in males and females.

## Materials and Methods

### Ethics Statement

The studies were carried out in strict accordance with the recommendations in the Guide for the Care and Use of Laboratory Animals of the National Institutes of Health. The protocol was approved by the Florida State University's Animal Care and Use Committee as protocol # 9028. All recovery surgery was performed under ketamine/xylazine anesthesia. Urethane anesthesia was used for terminal procedures. All efforts were made to minimize suffering.

### Animals

Female Sprague-Dawley rats who weighed 175–225 g at the beginning of the study were used. Rats were housed individually in a temperature-controlled room (22.2°C) in plastic cages lined with chip bedding, with ad libitum access to rat chow and water, and maintained on a 12-h light/dark cycle, with lights on at 07:00 AM.

### Estrous stage determination

Estrous stage was determined by daily vaginal lavage performed approximately 2 h after lights on for all rats. Traditional nomenclature was used [43]. All rats maintained regular four-day estrous cycles throughout the study.

### Surgically-induced endometriosis

Rats in metestrus or diestrus were anesthetized i.p. with a mixture of ketamine hydrochloride (73 mg/kg) and xylazine (8.8 mg/kg). Following aseptic precautions, an abdominal incision was made to expose the uterus and a 1–1.25 cm segment of the left uterine horn and associated fat tissue were removed and placed in warm saline. Four approximately equally-sized pieces of uterine horn (as close as possible to 2 mm×2 mm) were cut from this segment. Beginning at the caecum, these pieces of uterine horn were sewn on alternate cascading mesenteric arteries that supply the small intestine. After assuring hemostasis, the incision was sutured closed in layers, and butorphanol given (1 mg/kg, s.c.) as necessary. Rats were not tested behaviorally for the first post-operative week after surgery.

### Surgical cyst removal or sham-cyst removal

Rats in which transplant surgery had occurred 1 to 4 weeks earlier and were in diestrus, metestrus, or estrus, were anesthetized and abdominal organs exposed as for the original transplant surgery. The caecum was located and the transplants identified. Each cyst was carefully freed from surrounding fat and connective tissue and measured. Cysts were either removed completely (including the suture) with Castroviejo micro-dissecting scissors and a fine-tipped cautery (n = 8 rats), or, for sham-cyst removal, left in place (n = 4 rats). Post-operative procedures were the same as for the endometriosis surgery.

### Behavioral testing procedures

All behavioral training and testing was done between 3 and 8 h after lights on using techniques described previously [44]. Briefly, rats were first trained to terminate (“escape” from) vaginal distention produced by an inflatable balloon. The vaginal distention was produced by inflating a latex balloon (10 mm long×1.5 mm wide when uninflated) tied to a thin catheter with silk suture. The uninflated balloon was lubricated (K-Y jelly) and inserted into the mid-vaginal canal, located so that it would not touch the cervix even when inflated. Inflating the balloon with different volumes of water using a computer-controlled pump distended the vaginal canal. The pressure produced by each volume of distention (corrected for compliance characteristics of the balloon) was measured through a small-volume Cobe pressure transducer. After rats were trained to make the escape response, sessions to assess vaginal nociception were begun. During each assessment session, eight distention volumes were delivered three times each in random order at 60±15 sec intervals. Test sessions were run 3–4 times/wk on non-consecutive days. Testing sessions in each of the two assessment periods included at least 3 days in each estrous stage. Here, we show data obtained during proestrus, the stage in which the severity of vaginal hyperalgesia or referred muscle hyperalgesia is greatest [7]–[9].

### Experimental and control groups for the two behavioral studies

The first behavioral study assessed the time course of the development of vaginal hyperalgesia after transplant surgery in 10 rats. Vaginal nociception was calculated when the rat was in proestrus for 6–8 weeks before surgery to establish a baseline, then after transplant surgery for 12 weeks. The second behavioral study assessed the effects of removing the cysts prior to the development of functional innervation or the development of significant hyperalgesia (i.e., 1–4 weeks after transplant surgery) in eight rats. As a control, a sham cyst-removal surgery was performed in 4 rats during this same period. In this second study, vaginal nociception was calculated for 6–8 weeks before transplant surgery to establish a baseline then, after cyst-removal or sham cyst-removal surgery, for an additional 10 weeks.

### Calculation of vaginal nociception and hyperalgesic severity

Calculations were identical to those used in previous studies [9]. For each rat, the percent escape response to each distention volume was assessed in each session and averaged for each chronological test period. This procedure produced a graph of the percent escape response as a function of distention volume for each test period (Fig. 3). For each graph, an area-under-the-curve (AUC) calculation was performed using standard methods. The AUC calculation yielded a single value that described vaginal nociception during that test period.

In the first behavioral study (Fig. 1A), testing periods included the baseline period before transplant surgery and then weekly periods for 12 weeks post-transplant. The severity of vaginal hyperalgesia that developed after transplant surgery (i.e., the post-transplant change in vaginal nociception) was quantified by subtracting the baseline (pre-transplant) AUC value from each of the post-transplant AUC values at weekly time points, beginning with the second week. No data are available for the first post-transplant week because rats were not testing during this recovery period. The severity values were averaged across rats for each time point. Statistical assessment included a one-way ANOVA, which was significant (F_10,72_ = 4.37, *p*<0.001), and therefore followed by post-hoc LSD tests, with significance set at *p*≤0.05).

In the second behavioral study (Fig. 3B, 3C), there were two test periods (Fig. 3A): baseline (pre-transplant) and one post-transplant period that included the time from 5–10 weeks after transplant. Graphs of escape response as a function of volume were averaged across rats in each group (cyst removal group versus sham cyst removal group) for each test period (pre-transplant baseline and post-transplant). These data were statistically analyzed by a three-way repeated measure ANOVA (distention volume, test period, cyst surgery type), which was significant (F_31,160_ = 150.52, *p*<0.001), with significant (*p*<0.05) effects of volume, pre- versus post-transplant test period, surgical group, and all interactions (volume*test period; volume*surgery group; test period*surgery group, and volume*test period*surgical group). AUC vaginal nociception values for each surgical group and test period were also averaged across rats. The AUC values were statistically analyzed by a two-way ANOVA (surgical group, test period), which was also significant (F_3,20_ = 10.02, *p*<0.001), with significant effects of test period (*p*<0.001), surgical group (p = 0.028), and the interaction between test period and surgical group (*p* = 0.001). This overall significance pattern regardless of outcome measure (% escape or AUC) justified additional ANOVAs as appropriate, which were done using the AUC values.

### Immunostaining

Under urethane anesthesia (1.2 g/kg), rats in proestrus at 2, 3, 4, 6, or 10 weeks after transplant surgery (n = 3 to 5 rats at each survival time) were perfused with 4% paraformaldehyde. Cysts were harvested, post-fixed in 4% paraformaldehyde for 1 h and then incubated in 30% sucrose overnight. Cysts were embedded in Tissue-Tek Oct compound, frozen, cut serially in 20 µm-thick sections using a cryostat, and mounted on slides in 10 sets of sections (i.e., sections on a slide were separated by 200 µm). Adjacent sections from the same cysts were treated with 5% normal goat serum (NGS) for 1 h, then were immunolabeled with one of the two rabbit primary antibodies: either a marker for sensory C- fibers, calcitonin gene related peptide (CGRP, 1∶4,000; Chemicon, CA) or for sympathetic fibers, vesicular monoamine transporter 2 (VMAT2, 1∶1,000; Chemicon, CA) including 2% NGS at 4°C overnight. Sections were then incubated for 1.5 h with goat anti-rabbit Cy2-conjugated secondary antibody (1∶300, Jackson ImmunoResearch lab, PA). Negative controls were routinely performed for each immunostaining run; they included omission of the primary or the secondary antibody or both. No immunostaining of nerve fibers was evident in these controls. All immunostained sections were carefully examined using an epifocal microscope, and fiber labeling, if any, noted and qualitatively characterized. The representative sections shown here were photographed using an Optronics Microfire camera (Optronics International, Chelmsford, MA), and the entire image adjusted for contrast and brightness.

### Functionality of C-fiber innervation of the cysts

The Evans Blue dye (EB) extravasation method was used to assess neurogenic activity in intact and denervated cysts in rats at 1, 2, 3, 4, 6, or 10 weeks after ENDO surgery [13] (n = 8–12 rats for each survival time and each innervation class; i.e., intact or denervated). Previously, we observed that sensory fibers and postganglionic neurons for the sympathetic fibers that sprout into the cysts are located in the splanchnic nerve and coeliac ganglion, respectively [45]. Cyst denervation, therefore, involved removing these sources of innervation.

All rats were in proestrus on the day of the experiment for the 1- through 6-week survival time. We only had data at the 10 week-survival time from rats in metestrus. We thought it reasonable to include these 10-week data here, however, so a more complete comparison with the behavioral data shown in Figure 1 could be made, and because rats in metestrus, like those in proestrus, evidence vaginal hyperalgesia; i.e., the biggest difference in severity of hyperalgesia is between proestrus and estrus [7]. Rats were first anesthetized with urethane (1.2 g/kg) and placed on a warm heating pad (maintained as close as possible to 37° C). In the rats scheduled for cyst denervation, a laparotomy was performed to expose the splanchnic nerve and coeliac ganglion. The splanchnic nerve was identified and cut rostral to the coeliac ganglion, and the ganglion itself was carefully denervated by cutting all nerves associated with it. The abdominal muscles and skin were sutured, after which EB was injected. For EB injections, the jugular vein was exposed through an incision and catheterized. EB (50 mg/kg in saline) was then delivered through the catheter. Thirty min later, excess dye was rinsed out of blood vessels by delivering 200 ml of saline through the catheter. Cysts were harvested, weighed, and incubated in formamide at 60° C for 48 h. Optical density of the sample and standard solutions was measured spectrophotometrically (λ = 620 nm, UV: 1601; Shimadzu, Columbia, MD). The amount of EB extracted from each sample was calculated as mg EB/g of tissue. Because a two-way ANOVA was significant (F_11,120_ = 2.44, *p* = 0.009), data were analyzed by post-hoc unpaired t-tests, with significance set at p≤0.05.
